# Computing the Many-Body Green’s Function with Adaptive Variational Quantum Dynamics

**DOI:** 10.1021/acs.jctc.3c00150

**Published:** 2023-05-25

**Authors:** Niladri Gomes, David B. Williams-Young, Wibe A. de Jong

**Affiliations:** Applied Mathematics and Computing Sciences Division, Lawrence Berkeley National Laboratory, Berkeley, California 94720, United States

## Abstract

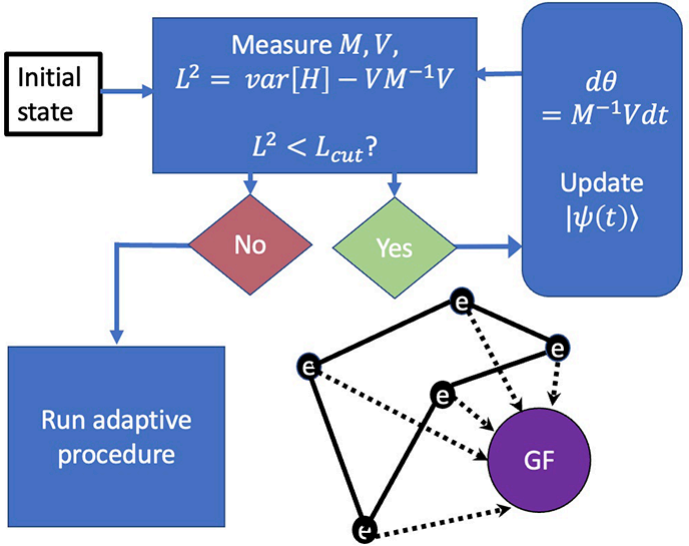

We present a method to compute the many-body real-time Green’s function using an adaptive variational quantum dynamics simulation approach. The real-time Green’s function involves the time evolution of a quantum state with one additional electron with respect to the ground state wave function that is first expressed as a linear–linear combination of state vectors. The real-time evolution and the Green’s function are obtained by combining the dynamics of the individual state vectors in a linear combination. The use of the adaptive protocol enables us to generate compact ansatzes on-the-fly while running the simulation. In order to improve the convergence of spectral features, Padé approximants are applied to obtain the Fourier transform of the Green’s function. We demonstrate the evaluation of the Green’s function on an IBM Q quantum computer. As a part of our error mitigation strategy, we develop a resolution-enhancing method that we successfully apply on the noisy data from the real-quantum hardware.

## 1 Introduction

The potential of quantum computers to solve scientific problems is many-fold.^1−8^ However, the state-of-the art quantum computers are still quite noisy,^9^ and it is an important research question to find ways to perform meaningful scientific calculations using them. Such interests have given rise to variational algorithms to study energy eigenstates and dynamics of spin and Fermionic systems.^10−16^ Looking beyond the evaluation of energy eigenstates, dynamical properties of electronic matter at low temperatures are of immediate interest to the scientific community. Electrons at low temperature experience strong Coulombic repulsion between one another, which poses a big challenge for computing their physical and chemical properties.^17−21^ Green’s function (GF) methods are a systematic way to study the such material properties. Despite the elegant power of the GF to efficiently predict a variety of electronic properties of materials, evaluating them exactly is equivalent to solving the full many-body problem which is impractical on even the largest supercomputers.^22^ In this work we explore a way to use quantum computers to overcome this challenge by computing a real-time GF using variational methods.

For the fault-tolerant quantum computers, direct computation of the GF in the frequency domain has been proposed using a preconditioned linear system method,^23^ the quantum Lanczos recursion method by exploiting a continued fractional representation of the Greens’s function,^24^ the Gaussian integral transformation,^25^ and a linear combination of unitaries.^26^ These methods, although showing advantage in the fault-tolerant regime, are not quite suitable for near-term applications. Most of the recent work on noisy intermediate-scale quantum (NISQ) simulation of the many-body GF is in the time domain via Hamiltonian simulation.^27−30^ Efficient Hamiltonian simulation can be done by doing Trotter decomposition of the time evolution operator. However, Trotter-based methods suffer from accumulating circuit depth with time and thus quickly become impractical for NISQ devices. Variational and other linear-algebra-based decompositions have been proposed to alleviate this issue. These approaches include simplification of time evolution unitary operation by applying Cartan decomposition,^28^ coupled cluster Green’s function method,^29^ and variational methods in real time^27,31^ or in the frequency domain.^32^ In spite of being variational, most of these methods still suffer from either large circuit depths or an ambiguity of a suitable ansatz. Adaptive approaches are known to provide scalable and compact ansatzes compared to fixed forms of ansatzes.^33,34^ In this work, by adopting the adaptive approach we obtain more compact and lower depth ansatzes lowering the depth of the quantum circuit.

Hamiltonian simulation to evaluate the real-time GF requires time evolution of a quantum state with an electron added to its ground state wave function. In other words, one needs a quantum state that requires application of a Fermion creation operator on the ground state to start with. When converted to spin operators, the creation operator is a linear combination of Pauli terms that should be applied to the prepared ground state. To prepare such a quantum state in the quantum computer is nontrivial. Time evolution of the this state has been done using McLachlan’s variational method for real-time dynamics^27,31^ using variational Hamiltonian ansatz (VHA).^35,36^ Accuracy of the variational solution is systematically increased by increasing the number of layers or depths of the ansatz. However, this poses a challenge for the near-term device since many numbers of layers are needed to reach the desired accuracy. Moreover, there exists ambiguity over how many numbers of layers should be used. As a result, the method may become highly nonscalable as can be seen for larger size calculations in previous works.^27,31^

Since we are interested in the time evolution of the state, we avoid the additional electron state preparation by expressing it as a linear combination of state vectors and apply McLachlan’s variational method for real-time dynamics. The rest of the paper is organized as follows. We first present a brief overview of Green’s function in correlated electronic systems and then present our modified McLachlan’s equation used to simulate real-time dynamics. We then discuss our adaptive strategy and present our preliminary ideal state vector results for the *N* = 4 Hubbard model followed by an estimation of resources and error complexity. Finally, we present results of a hardware run to demonstrate the applicability of the method on NISQ devices.

## 2 Method

### Green’s Function Overview

Given a time-independent Hamiltonian , the time evolution of the annihilation operator for a single particle quantum state *p* is given by . With a ground state |ψ_0_⟩ and energy *E*_0_, the retarded GF of the system can be then written in terms of the *G*^>^ and *G*^<^ Green’s functions as^19^
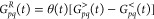
1where
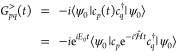
2
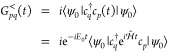
3

In the context of many-body physics, one is also interested in the Fourier transform of the Green’s function
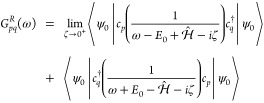
4where ω is the frequency and ζ is a small positive number used as a damping factor to make the Fourier integral convergent. We will compare our results for the Fourier transform of our real-time data with this formula.

### Algorithm

To obtain *G*^>^(*t*) in a near-term device, we first time evolve the state |ψ^*q*^⟩ = *c*_*q*_^†^|ψ_0_⟩ to get  and then find its overlap with the state |ψ^*p*^⟩ = *c*_*p*_^†^|ψ_0_⟩. *G*^<^(*t*) can be obtained similarly by starting from *c*_*q*_|ψ_0_⟩. A conventional VQE^12^ or its adaptive version^37^ can be used to prepare a variational state representing |ψ_0_⟩. Using the fact that the creation and annihilation operators can be expressed in terms of a sum of Pauli words using Jordan Wigner transformation, i.e, *c*_*q*_ = ∑_α_η_α_^(*q*)^*P*_α_, where η_α_^(*q*)^ are complex numbers and *P*_α_ are Pauli words, we can write down the initial state for our variational simulation |ψ^*q*^⟩ as a linear combination of multiple quantum states, each of which we denote as branches, i.e.
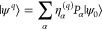
5where *P*_α_|ψ_0_⟩ is a branch state.

To simulate the dynamics of |ψ^*q*^⟩, for each branch state in |ψ^*q*^⟩ using variational methods, we will use the recently developed adaptive variational approach (AVQDS) .^33^ Our aim is to build an ansatz |Ψ[**θ**(*t*)]⟩, which is parametrized by a real time-dependent variational parameter vector **θ**(*t*), such that it represents |ψ^*q*^(*t*)⟩ up to a given accuracy. At any instant of time the ansatz can be written as
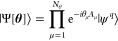
6where the *A*_μ_ are Pauli words. The variational form of eq 6 will accurately simulate the unitary evolution  by time evolving each of the branch states. It is important to point out one key difference of our approach to that described in ref (27). In their work, the time evolution operators  for |ψ_0_⟩ and *P*_α_|ψ_0_⟩ are approximated by the same unitary using VHA. In our case, we approximate the time evolution operator by a unitary  for the full *n* + 1-electronic state |ψ^*q*^⟩ using an adaptive protocol.

When using the variational method for a dynamics simulation, for a system described by a quantum state |Ψ⟩ evolving under a Hamiltonian , the time evolution of density matrix ρ = |Ψ⟩⟨Ψ| is given according to the von Neumann equation
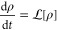
7with . In the McLachlan’s variational quantum simulation approach, the squared distance between the variationally evolving state and the exact propagating state is minimized. It is also called the McLachlan’s distance, which is defined as
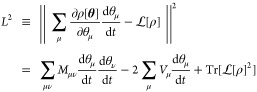
8Here  is the Fröbenius norm of the matrix ρ. The matrix *M* is real symmetric with elements defined as
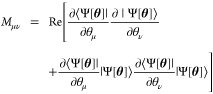
9The vector *V* is given by
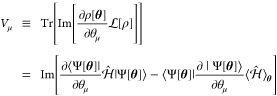
10where  and
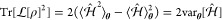
11which describes the energy variance of  in the variational state |Ψ[**θ**]⟩. The minimization of the cost function in eq 8 with respect to  leads to the following equation of motion for the variational parameters:
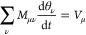
12and the minimum value of the McLachlan’s distance is given by
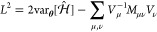
13With the initial state for time evolution as eq 5, elements of *M* and *V* can be written as a linear combination of terms that mixes the branch states during time evolution
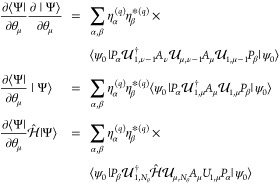
where . Similarly, the expectation value of any observable can be calculated as
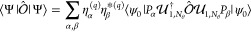
Note that each of the branch states is evolved by the same unitary  at every time step. All of the above quantities can be measured in a quantum device using a Hadamard test type circuit or a linear combination of unitaries (LCU) to reconstruct the elements of *M* and *V*.^33,38^ We will provide a detailed discussion and complexity analysis in a later section.

Under the adaptive scheme, McLachlan’s distance *L*^2^ is computed for a series of new variational ansatzes. Each new ansatz is composed of a product of  and the existing ansatz. The operator  is chosen from a preconstructed (fixed) operator pool of size *N*_op_ in such a way that gives the lowest *L*^2^. Given an existing ansatz with *N*_*p*_ parameters, as each operator is added to it, the dimension of **θ** increases from *N*_*p*_ to *N*_*p*_ + 1. Accordingly, the matrix *M* (eq 9) increases from *N*_**θ**_ × *N*_*p*_ to (*N*_*p*_ + 1) × (*N*_*p*_ + 1) and that of the vector *V* (eq 10) increases from *N*_*p*_ to *N*_*p*_ + 1. In this way, the ansatz is dynamically expanded by including additional operators to maintain the McLachlan distance below a certain threshold *L*_cut_.

The differential equation of motion (eq 12) is then numerically integrated to obtain the dynamics at each time step.
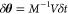
14With *δt* as the time step size, the global truncation error over the total simulation period scales linearly with *δt*. The error from numerical integration can be lowered by choosing a smaller step size (*δt*). In this work we have used the Euler method, although alternative approaches using Runge–Kutta can also be used.^39^

It is important to emphasize here that, except for the measurement of *M* and *V*, all the other calculations in our computation are done using a classical processor. When we measure *M* and *V* from the quantum processor, there may be shot noise which can cause a high condition number and inconsistency in measured *M*. Reference (40) has shown that the error in δ**θ** increases proportionally with *M*^–2^. The presence of shot noise in *M* exacerbates the error. To deal with these issues of large condition number, we can avoid matrix inversion while solving eq 12 by minimizing the cost function and disregarding very small singular values. This approach can help reduce the irregularities caused by shot noise. We do this by avoiding solving the equation of motion, eq 12, and using optimization of the cost function .

Finally, for the Green’s function, we first measure
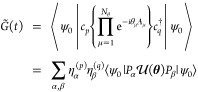
15Clearly, from eq 2, a product of  and *G̃*(*t*) will give us *G*^>^. An exactly similar strategy can be followed to compute *G*^<^. The term  can be measured using a standard Hadamard test-like circuit or its variants^41^ or the Hadamard overlap test shown in Appendix A.

### Padé Approximation

One drawback of a real-time approach for finding frequency domain observables is long simulation times (*T*) required for a converged spectrum. As the system size grows bigger, the proximity of the density of states makes it harder to resolve the spectral without longer simulations due to the Fourier uncertainty principle.

By making use of Padé approximants, we may decrease the simulation time and accelerate the convergence of the Fourier transform of the time-dependent GF. The method has been successfully applied to accelerate the computation of broadband absorption spectra in quantum chemistry.^42^ The method of Padé approximants equates the initial power series of  (expressed in its discrete form with time step *δt*) to a ratio of power series expansions
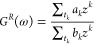
16where *t*_*k*_ = *kδt*, and . The coefficients *a*_*k*_ and *b*_*k*_ can be obtained by solving .^42^ The expression of *G*^*R*^(ω) as a rational function allows for its evaluation at arbitrary spectral resolution, in contrast to the *fixed* spectral resolution yielded by the fast Fourier transform (FFT). This phenomena can also be thought of as an extrapolation of *G*^*R*^(*t*) sampled on [0, *T*] to *t* → *∞*. Naturally, the validity of this approximation depends on the spectral modes which are sufficiently sampled during the simulation time interval. We discuss simulation parameter selection in Appendix C.

## 3 Results

We have applied our method to evaluate the GF for the one-dimensional Hubbard model with open boundary conditions
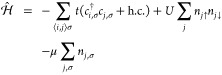
17To preserve particle–hole symmetry, we choose μ = *U*/2. The value of the hopping parameter *t* is chosen to be unity. The Hamiltonian is mapped to qubits using Jordan Wigner transformation. Throughout the rest of the paper, we consider the Hubbard model at half-filling with total spin and its “*z*” component (*S*_*z*_) to be zero, i.e, the number of electrons the same as the number of lattice sites, and *N*_*↑*_ = *N*_*↓*_ and open boundary conditions.

### Ground State Preparation

The ground state |ψ_0_⟩ is prepared using the adaptive variational imaginary time evolution (AVQITE) approach.^34^ The method is based on McLachlan’s variational principle applied to imaginary time evolution of variational wave functions. The variational parameters evolve deterministically according to equations of motions that minimize the difference to the exact imaginary time evolution, which is quantified by the McLachlan distance. Rather than working with a fixed variational ansatz, where the McLachlan distance is constrained by the quality of the ansatz, the AVQITE method iteratively expands the ansatz along the dynamical path to keep the McLachlan distance below a chosen threshold. We denote time along the imaginary axis by τ. In our calculation, we have chosen the threshold to be 1.0^–4^ and a time step size 0.01 such that after *k* time steps τ = *k*Δτ. The operator pool in any adaptive method plays a crucial role. In our AVQITE method, we have used a qubit adapt pool proposed by Tang et al. .^43^ Under this scheme, the pool operators are defined as
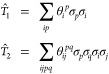
18where σ_*p*_ can be *X*_*p*_ and an odd number of *Y*_*p*_’s only. We use the individual terms from eq 18 as the operators in our pool. While there are multiple choices of operator pools, variational ansatzes generated with qubit adapt pools are much shallower.^34^ Despite the fact that utilizing a qubit adapt pool results in an increased number of terms in the operator pool, which subsequently requires more measurements to perform the adaptive procedure, the primary limitation of NISQ devices is the circuit depth. Therefore, the focus of this work is primarily on minimizing the circuit depth rather than the number of measurements required.

**Figure 1:**
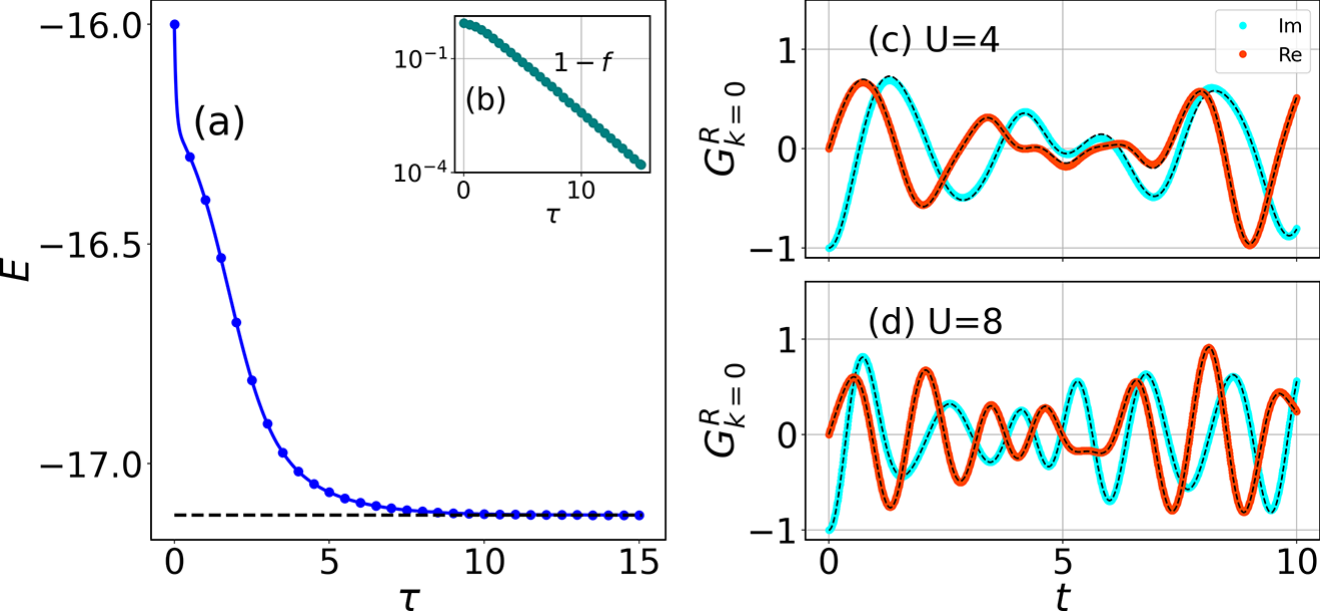
Ground state preparation and real (imaginary) part of the retarded GF for the *N* = 4 Hubbard model. (a) Ground state energy convergence as a function of time τ along the imaginary axis, at half-filling for *U* = 8.0. (b) Ground state infidelity is defined as 1 – |⟨Ψ(**θ**(τ))|Ψ_exact_⟩|^2^. (c) and (d) Real and imaginary parts of the time-dependent retarded GF for *U* = 4.0 and *U* = 8.0. Exact results are shown in black curves.

Our imaginary time evolution starts at τ = 0 from a product state with the upspin electrons and the downspin electrons being segregated at the left and the right segment of the lattice, respectively. For a four-site model, such an arrangement would look like |*↑↑↓↓*⟩. A sample result for the ground state calculation is shown in Figure 1, for the *N* = 4 site Hubbard model with four electrons. The time evolution τ conserves *S*_*z*_ and the total number of electrons. In Figure 1a, AVQITE (shown in the blue curve) converges to the ground state after τ = 8 with an infidelity lower than 10^–4^. We define the infidelity as 1 – |⟨Ψ(**θ**(τ))|Ψ_exact_⟩|^2^, where |Ψ_exact_⟩ is the ground state from exact diagonalization.

Alternatively, variational ways can be adopted to prepare *c*_*q*_^†^|ψ_0_⟩ following a method originally proposed to simulate generalized time evolution^44^ or the method described in ref (45) by starting from a state with *n* + 1 particles. Following ref (44), the algorithm is based on converting the static algebraic problem into a dynamical process, evolving the initial vector |ψ_0_⟩ to the target state *c*_*q*_^†^|ψ_0_⟩. The evolution path is via a linear extrapolation, , where  and  is the identity. According to ref (45) , *c*_*q*_^†^|ψ_0_⟩ is constructed by optimizing an ansatz with *n* + 1 particles. The main idea of this work is approximating the time evolution operator e^–*iHt*^ by a unitary  for the state |ψ_*q*_⟩; hence, as long as the initial state is |ψ_*q*_⟩, the circuit depth for  should remain the same as presented in this work. However, variationally preparing |ψ_*q*_⟩ will require another set of unitary operations, which could increase the circuit depth.

### Real-Time Simulation

Using the ground state of the Hamiltonian obtained from AVQITE, we now simulate the dynamics of |ψ^*q*^⟩ using AVQDS. For implementation in the real device, the system Hamiltonian  and the Fermionic creation and annihilation operators *c*_*q*_^†^, *c*_*q*_ are expressed as a linear combination of Pauli terms using Jordan–Wigner transformation. Like any other adaptive methods, the choice of an operator pool plays a crucial role here. In our current work we use the so-called Hamiltonian pool along with the additional terms in the Fermionic creation and annihilation operators.^33^ Since the time evolution of |ψ_*q*_⟩ is nothing but the Hamiltonian simulation of a quantum state, the natural choice is the so-called Hamiltonian pool.^33^ In a Hamiltonian pool, the system Hamiltonian is first transformed to a qubit representation for calculations on QPU. The operator pool  is constructed from only those Pauli terms that appear in the system Hamiltonian in the qubit representation. In order to incorporate one additional electron in |ψ_*q*_⟩, we include additional terms arising out of the qubit representation of *c*_*q*_^†^. Similar to the qubit adapt pool for imaginary time evolution, such breakdown of the Hamiltonian into Pauli terms is able to generate low-depth circuits. Since *c*_*q*_^†^ is generally the sum of a unitary and an antiunitary term, the size of the operator pool is increased by a factor of two; hence, the operator pool roughly scales as the number of terms in the Hamiltonian.

The GF can be computed for different pairs of sites within a lattice. For a more compact representation, we present our results in momentum space using a linear combination of all pairs of real-space GF
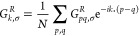
19where *k* is the momentum and *p*, *q* are lattice site indices. The results for ideal noiseless real-time evolution of the imaginary part of *G*_*k* = 0_^*R*^ for *U* = 4 and 8 are shown in Figure 1c and d. The threshold of the McLachlan distance depends on multiple factors, such as the initial state, the Hamiltonian, and the choice of the operator pool. Depending on the initial state, the operators in the pool are not sufficient to the lower McLachlan’s distance beyond a certain limit. This may lead to a slightly different *L*_cut_^2^ for the different initial states. In order to obtain an estimate of *L*_cut_^2^ in the beginning, we run the adaptive procedure only once starting from no parameters in the ansatz and check the value of the lowest *L*^2^ distance given by eq 13. We set this value as our *L*_cut_^2^ for the rest of the calculation. We provide the value of *L*_cut_^2^ for different pairs of sites in Table 1.

**Table 1:** *L*_cut_^2^ for *N* = 4 Calculation for a Given *U* and a Pair of Lattice Points (*p*, *q*)

*U*; (*p*, *q*)	(0, 0)	(0, 1)	(0, 2)	(0, 3)	(1, 1)	(1, 2)
4.0	10^–3^	10^–3^	10^–3^	10^–3^	5 × 10^–2^	5 × 10^–2^
8.0	10^–3^	10^–3^	10^–3^	10^–3^	6 × 10^–2^	6 × 10^–2^

We run our simulation for a total time of *T* = 10 with *δt* = 0.01. The black dashed lines show the exact result, and the cyan and orange represent the variational results for the imaginary and the real part of the Green’s function, respectively. The figure readily shows that the exact and the variational results are a very good match.

**Figure 2:**
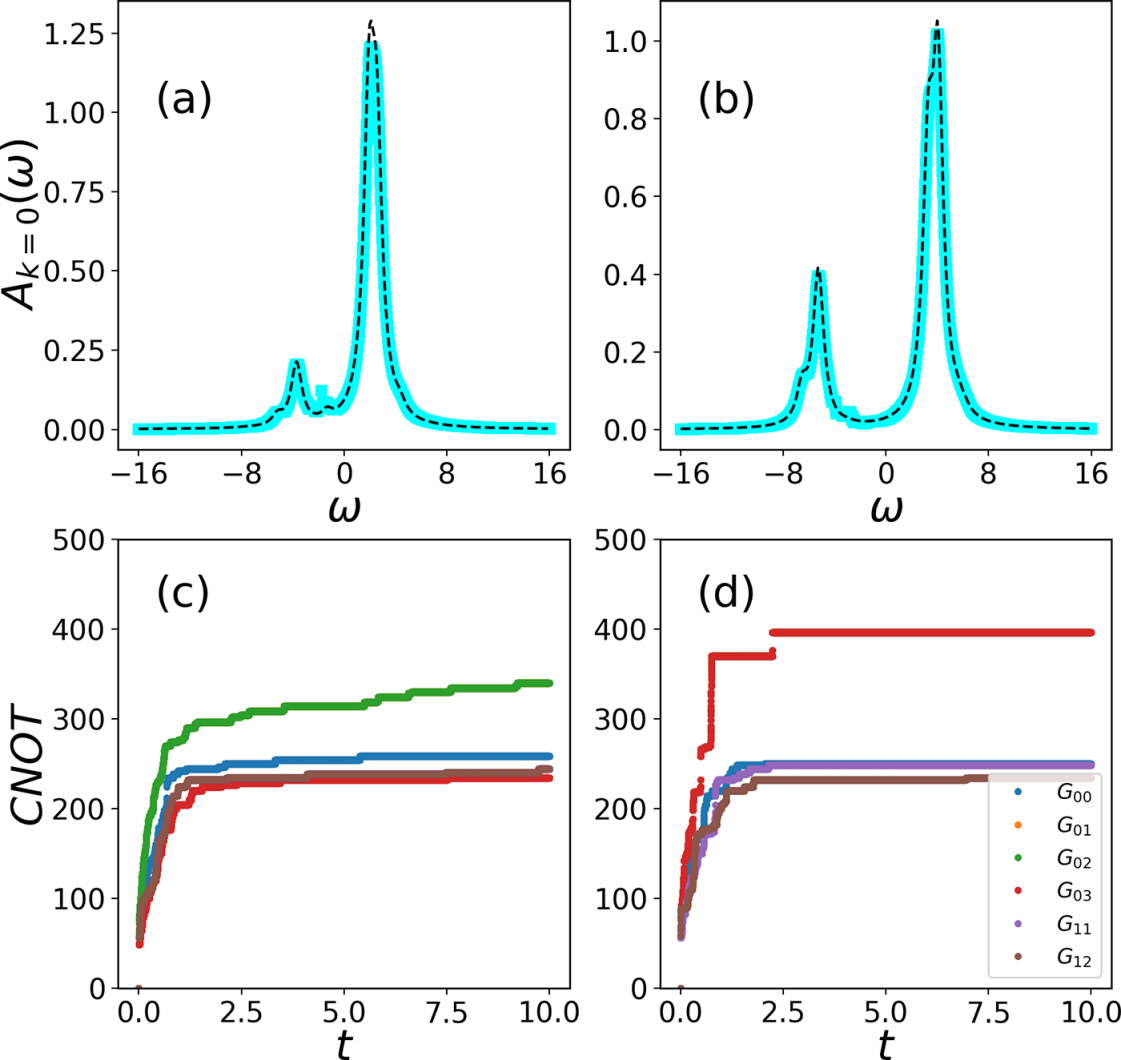
Spectral function Hubbard model at half-filling for *N* = 4, *U* = 4.0, and *U* = 8.0 (a and b). The blue curves are obtained by applying the Padé approximation to the real-time evolution data. Exact results are computed using eq 4 and are shown in black. The spectral function is obtained by Fourier transforming the data in Figure 1c and d. Panels c and d shows the upper bound of the number of CNOTs needed to simulate the real-time GF for *U* = 4 and 8.

Next, using the real-time data, we find an approximate Fourier transform of the real-time data using the Padé approximation. The imaginary part of the Fourier transform of *G*^*R*^ is also called the spectral function . We show plots of *A*(ω)_*k*=0_ in Figure 2a and b for *U* = 4 and 8, respectively. The blue and black curves represent the AVQDS and the exact calculations. Since the time series is not convergent, one needs to add a small damping factor to obtain a converged Fourier transform. We have used a damping of ζ = 0.5 for each of the plots in Figure 2a and b. The results clearly shows an excellent match of our method with the exact results from eq 4. The real advantage of using the Padé approximation is that, in order to obtain a convergent Fourier transform, we need a real-time simulation for a total time that is an order of magnitude smaller than the existing calculations in the current literature.^27,28,45^

### Resource Estimation and Complexity Analysis

**Figure 3:**
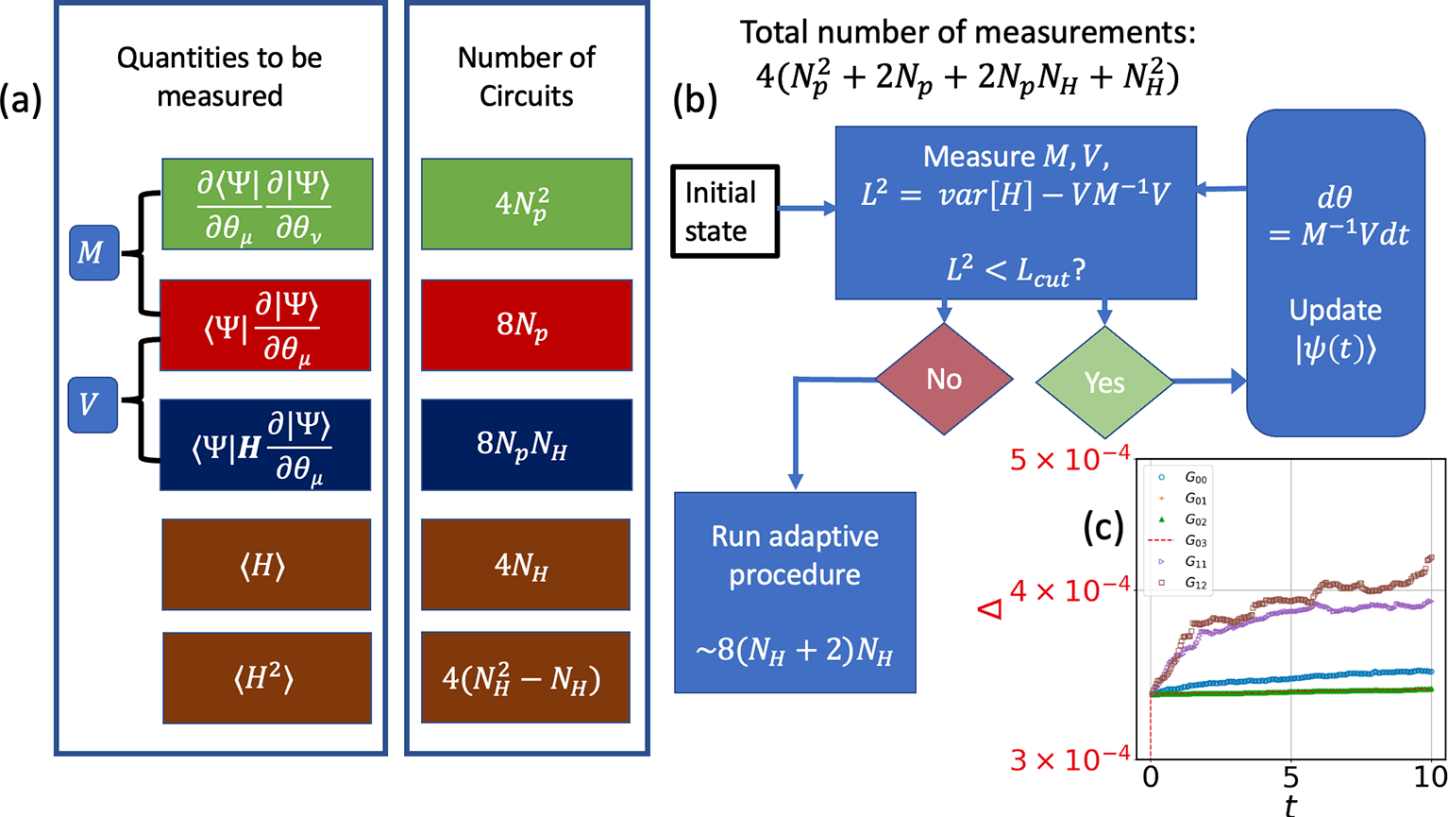
Resource estimation and error analysis of AVQDS for Green’s function evaluation. *N*_*H*_ and *N*_*p*_ are the number of terms in the qubit-transformed Hamiltonian and the number of parameters in the ansatz, respectively. (a) Number of measurements required to evaluate each term in the dynamics. (b) Flowchart of the dynamical simulation. The operator pool will contain *N*_*H*_ + 2 number of terms. (c) Error (Δ) for unitary generation in the ansatz vs time for different sets of lattice sites (*p*, *q*) for the *N* = 4, *U* = 4 Hubbard model. Each of the real-space GFs is finally combined to get *G*_*k*_.

We provide a resource estimate of our described method in this section. We assume Jordan Wigner (JW) transformation of the Fermionic operators to provide the estimate. Under the JW scheme, *c*_*i*_^†^ and *c*_*j*_^†^ will have two Pauli terms each. If there are *N*_*H*_ number of terms in the qubit-transformed Hamiltonian, the Hamiltonian operator pool will contain *N*_*H*_ + 4 number of terms. The additional four terms arise from the qubitized version of *c*_*i*_^†^ and *c*_*j*_^†^. In the case of the diagonal terms of the GF, the operator pool size will be *N*_*H*_ + 2. The leftmost column in Figure 3a shows the quantities that are required to measure the time evolution, which will be combined to calculate *M* and *V* in eq 12. To measure each term, the number of circuits required with an ansatz with *N*_*p*_ parameters is given in the right column. Combining all of them, the algorithm requires 4(*N*_*p*_^2^ + 2*N*_*p*_ + 2*N*_*p*_*N*_*H*_ + *N*_*H*_^2^) circuits to be run for each time step. There will be an additional circuit run of 8(*N*_*H*_ + 4)*N*_*H*_ in the case in which the method enters the adaptive procedure.

Once the variational parameters for each time step are obtained, separate circuits could be run to measure the GF. Since our method has found the unitary that represents the time evolution of |ψ^*q*^⟩, we just need to run four circuits to measure the real and imaginary parts of *G*_*pq*_^>^(*t*) at each time step. In other words, we need to run a circuit to measure  for each term in eq 15. In this work, since we are dealing with a particle–hole symmetric Hamiltonian, the time-reversed partner *G*_*pq*_^<^(*t*) will be just a complex conjugate of *G*_*pq*_^>^(*t*). Use of such symmetries makes the additional dynamics simulation of *G*_*pq*_^>^(*t*) redundant.

In order to estimate an upper bound for the number of CNOT gates (*N*_*X*_) required at each time step, we first estimate the number of CNOTs in the ansatz. Since the ansatz consists of unitaries of the form , where *P*_*l*_ is a Pauli word of length *l*, the number of CNOTs in the unitaries is given by *∑*_*l*_2(*P*_*l*_ – 1). To implement a controlled unitary, we need two additional CNOTs from an ancilla qubit. Therefore, for an ansatz with *N*_*p*_ parameters, the total number of CNOTs is given by *N*_*X*_ = *∑*_*l*_2(*P*_*l*_ – 1) + 2*N*_*p*_. Figure 2c and d shows the upper bound of the number of CNOT gates needed to compute the real-space pairwise GF for *U* = 4 and 8, respectively.

In order to estimate error due to AVQDS and compare it with Trotter-like methods, we first consider the error due to approximating the time evolution by the series of unitaries. To quantify these errors, we calculate  at every time step, where  approximates is a unitary in either variational or Trotter-like methods. We show in Figure 3c the variation of Δ for our variational and Trotter approach using the red and black curve, respectively. The error Δ arises from the approximation adopted in the respective methods when no external noise is present and an infinite number of measurements is assumed. Figure 3c shows that the variational errors are of the order (Δ ∼ 4 × 10^–4^). Considering this negligible amount of error, the saving in terms of the number of unitaries using AVQDS is huge. To see this, consider the number of unitaries required for Trotter-based methods that scales as .^46^ For the case of the *N* = 4 site Hubbard model (8 – qubit), the number of unitaries required for Trotterization with the above Δ and *N*_*H*_ = 17 would be ∼3 × 10^7^.

We also benchmark our method against VHA presented in ref (27). For *N* = 2, *U* = 4 Hubbard model, our method saturates at *N*_*p*_ = 4 parameters in the ansatz {*Y*2*Z*3*Y*4, *Z*3*Z*4, *Y*2*Z*3*Y*4, *Z*3*Z*4} requiring 12 CNOTs only for the unitary. According to ref (47), the number of CNOTs for a single layer of VHA for the Hubbard model scales as 8*N*^3/2^ + *N* – 4 *N*^1/2^. *N* = 2 with 8 layers and *N* = 4 with 16 layers of VHA^27^ will, therefore, require about 150 two-qubit gates and 960 gates, respectively. Both these numbers are much larger than our upper bound of CNOTs for *N* = 2 and 8, as can be seen from Figure 2c and d. It is also worth noting that VHA with 16 layers for the *N* = 4 case does not show satisfactory accuracy for long-time simulation. Clearly, AVQDS is much more efficient than the Trotter-based method and more resource efficient than VHA. A recent work^31^ has deployed symmetries within the VHA scheme to calculate a GF that has reduced the circuit depth. Their strategy might by combined with our adaptive method to reduce the cost of the adaptive procedure and thereby reduce the multiqubit gate count.

## 4 Hardware Results

In order to demonstrate our algorithm in a near-term quantum computer, we store the classically computed parameters of the time evolution on a disk and use them to compute the GF at the respective time step. This amounts to running the circuits for eq 15 in IBM’s 27-qubit processor ibmq_kolkata based on the Falcon architecture. To run the algorithms successfully, careful compilation of the prepared circuit based on the selected quantum device is required, and error mitigation strategies need to be applied to get reliable results.

### Circuit Generation

Multiple circuits were generated with the Qiskit transpiler. The transpiler stochastically adds swap gates to the circuit and therefore produces multiple circuits with variable numbers of CNOTs. We choose the circuit that has the lowest number of CNOTs. Using these circuits as a base, we compile the best circuit with an open source toolkit, the Berkeley Quantum Synthesis Toolkit (BQSKit).^48^ BQSKit combines state-of-the-art partitioning, synthesis, and instantiation algorithms to compile circuits and was able to reduce the number of CNOTs by 30–40 percent. Finally, we use the standard tools in Qiskit to add dynamical decoupling by implementing periodic sequences of gates, which is intended to average out unwanted system–environment couplings of the idle qubits.^49^

### Error Mitigation and Postprocessing

Readout or measurement error mitigation was done on IBM Quantum systems in a scalable manner using the matrix-free measurement mitigation (M3) package.^50,51^ M3 works in a reduced subspace defined by the noisy input bitstrings that are to be corrected. We have used this package to apply readout error mitigation.

A peak-sharpening algorithm^52^ was applied as a postprocessing error mitigation approach. The approach builds on the observation that the histogram of the bitstrings of the noisy data is flatter than the noise-free data. Clearly, a sharper distribution of the bitstrings will lead to a better estimate of the observables. To this end, the peak-sharpening algorithm applied to the bitstring data artificially improves the apparent resolution of the peaks.^52^ Details about the method are provided in Appendix B. Application of this method significantly improves our results.

### Results

**Figure 4:**
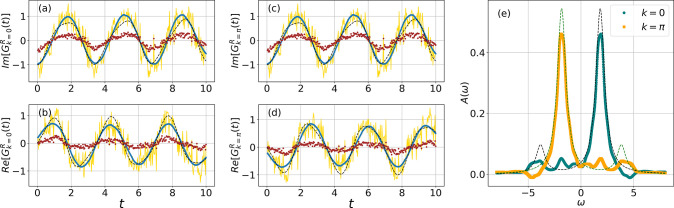
Device simulation run of the dynamics of Green’s function (a–d) before (brown) and after (gold) applying resolution enhancement. The teal curve (final error-mitigated data) is obtained by applying noise filtering of the “gold” data. The experiments are run on ibmq_kolkata with 100 000 shots. Panel (e) shows the Padé approximation applied to the error-mitigated data. Teal and orange are for *k* = 0 and π, respectively. The exact results are shown in dashed curves for comparison. The model chosen is the *N* = 2, *U* = 4 Hubbard model.

We run our simulation for four qubits (that is, the *N* = 2, *U* = 4 Hubbard model). The results are shown in Figure 4. In order to generate the data in Figure 4a–d, we run two sets of experiments to compute *G*_00_^>^(*t*) and *G*_01_^>^(*t*). The “less than” GFs are obtained from the “greater than” by exploiting the particle–hole symmetry . We then combine them to obtain retarded GFs in momentum space using eq 1 and eq 19. In Figure 4a–d we present the real and imaginary parts of the *G*^*R*^(ω) for momentum *k* ∈ {0, π}. The exact time evolution data is shown in the black dashed curve. The original noisy data is plotted in brown. It already has error-suppression effects (described earlier) applied to the circuits. As a postprocessing step, we apply the resolution enhancement method on the brown data to obtain the gold data. Although wiggly, the “golden” curve fluctuates around the exact results. So we apply an additional Savitzky–Golay filter on the data to obtain a smoother curve that is shown in teal.

The spectral function is calculated from the time evolution data using the Padé approximation. The plot is shown in Figure 4e. The exact *A*(ω) is shown in black, and the device results are shown in teal. Reference (31) uses prior knowledge of exact results^53^ to estimate the total simulation time (*T*). On the other hand, ref (27) has shown that much longer *T* is required to get a good estimate of the Fourier data. Use of the Padé approximation avoids the ambiguity of the magnitude of *T* and enables us to obtain reliable Fourier-transformed data using a much smaller simulation time.

## 5 Summary

Using a combination of McLachlan’s variational principle for quantum dynamics and an adaptive strategy, we have shown a method for calculating a many-body Green’s function in a near-term quantum computer. The real-time Green’s function is transformed into Fourier space by the use of the Padé approximation. The use of the approximation helps avoiding long-time dynamics simulation. Our method requires an accurate estimate of the ground state energy *E*_0_. For example, *E*_0_ can be accurately estimated from any other state preparation methods (phase estimation, imaginary time evolution). We have applied the method to compute Green’s function for the 1-D Hubbard model at half-filling. Our result shows a good match with the exact results. By using classically precomputed parameters, we compute the real-time Green’s function for a two-site Hubbard in a real quantum computer and apply multiple error suppression and error mitigation strategies that give satisfactory results.

Our method can be extended to compute Green’s function for other quantum-chemical systems and the two-particle Green’s function to compute response functions. The key advantage of using an adaptive approach like AVQDS is they can generate much smaller and compact ansatzes with an optimal number of parameters compared to other variational and Trotter-based methods. AVQDS has shown “saturation”-like behavior in terms of the parameters (and CNOT) in other calculations.^33,54^ The saturation of the number of parameters (and the number of CNOTs) is a feature of adaptive methods and can be exploited for a diverse class of physical systems. The error mitigation strategy presented in this paper is novel and can be applied as a new postprocessing scheme to other measurements in the NISQ devices. We will investigate this method more in our future research for error mitigation schemes. The experimental data shows the potential of the quantum computers for nontrivial scientific applications and would encourage further investigation of correlated many-body systems in quantum computers.

## Figures and Tables

**Figure 5:**
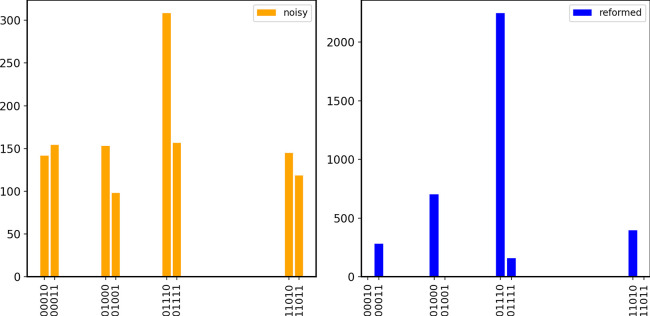
Histogram of bitstrings of noisy simulation (a) before and (b) after applying resolution enhancement.

**Figure 6:**
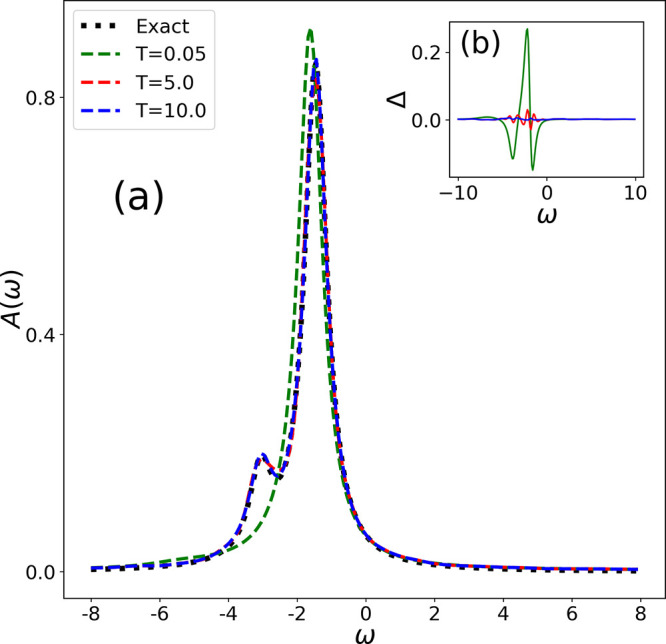
(a) Spectral function vs frequency for different simulation runtime *T*, for the *N* = 2 Hubbard model. (b) Difference (Δ) between the exact and approximate spectral functions for the corresponding data in plot (a). Δ becomes smaller as we move from *T* = 0.05 (aqua) to *T* = 10 (blue curve).

## Appendix A

In order to measure , let us consider the transformation of |ψ_0_⟩ ⊗ |0⟩ in the following circuit

The probability of measuring the qubit 0 to be in state |0⟩ and |1⟩ is

20

21

Hence the desired expectation value is

22

## Appendix B

Our resolution enhancement based noise reduction approach primarily assumes that the bitstring data generated in a noisy experiment loosely follow the ideal probability distribution of the bitstrings. In other words, noise and measurement errors lead to a flatter histogram but retain the true behavior of the histogram. In order to approach the ideal bitstring distribution, we make use of resolution enhancement measures commonly used in image processing.^52^

Calling *y*_*j*_ the frequency of the noisy data of the *j*th bitstring, resolution-enhanced frequency is obtained using *r*_*j*_ = *y*_*j*_–*k*_2_*y*_*j*_^″^, where *r*_*j*_ is the reformed frequency and *y*_*j*_^″^ is the second derivative of the noisy data w.r.t the decimal representation of the bitstrings. The parameter *k*_2_ can be modified to tune the resolution of the final data.

The weighting factor *k*_2_ can be chosen based on what gives the best trade-off between resolution enhancement, signal-to-noise degradation, and baseline flatness. The optimum choice depends upon the width, shape, and digitization interval of the bitstrings. After obtaining *r*_*j*_ we identify the bitstrings nearest to the peaks from the resolution-enhanced data. We then switch back to the binary representation of the bitstrings and replace the *y*_*j*_’s by the *r*_*j*_’s.

In order to avoid the ambiguity of the optimal value of *k*_2_, we iterate over several values of *k*_2_ and calculate the probability *p*_0_ of the ancilla qubit for each of them. We continue iterating until *p*_0_ converges with a certain threshold ϵ. In other words, if *p*_0_(*k*_2_^(*j*)^) is the probability at the *j*th iteration, we first calculate the average of *p*_0_(*k*_2_^(*j*)^) over the previous *j* values of *k*_2_. Thus, we may define

23

We stop the loop if . For our calculation we chose ϵ ∼ 10^–4^ and varied *k*_2_ in the range [0, 4] in steps of 0.1. To understand the effect of the method, we show the results in Figure 5, where the original noisy data (after applying Fermion number conservation) is shown in the left panel and the right panel shows the data after the resolution enhancement is applied. It can be clearly seen that the histogram in the right panel has sharper peaks.

## Appendix C

The ability of the Padé approximation to produce a faithful representation of the Fourier transformation depends the sampling rate of important spectral modes of a particular time signal. As high-frequency modes are more often sampled than low-frequency modes in any particular time interval, their spectral convergence is rapid and practically limited primarily by the Nyquist frequency determined by the time step. This phenomenon has been demonstrated on a number of occasions in the simulation of X-ray absorption spectra for molecular systems.^55−57^ For low-frequency modes, a sufficient *T* must be chosen to ensure that the desired spectral components are sufficiently sampled. This latter challenge is highly problem dependent and depends on the spectral characteristics of *H*. In cases where an insufficient *T* is chosen, the Padé approximation will contain both physical and unphysical peaks, the latter of which may be understood as a sinc-convolution of the physical signal arising from early truncation of the *G*^*R*^(*t*). The presence of these peaks is known to disappear rapidly with growing *T*,^42^ and they are thus identifiable through simple convergence analysis on the resulting *G*^*R*^(ω) at several values of *T*.

In this section, we show how unphysical peaks in the Padé approximation can vanish by running longer time simulation for the dynamics studied in this work. In Figure 6, we show a plot for *A*(ω) calculated for three different *T*. The results are for the *N* = 2 Hubbard model with *U* = 4.0, and *A*(ω) has been obtained from *G*_00_(*t*). In Figure 6a, it can be seen that as we increase *T* from 0.05 to 10.0, the variational results (blue curve) start matching well with the exact results (shown in black). For better clarity, in Figure 6b, we plot the error Δ = *A*_exact_(ω) – *A*_variational_^Pade^(ω) for different *T*. Clearly, Δ becomes lower for larger *T* making the results more reliable.
